# Prospective phase-II-study evaluating postoperative radiotherapy of cervical and endometrial cancer patients using protons – the APROVE-trial

**DOI:** 10.1186/s13014-017-0926-5

**Published:** 2017-11-28

**Authors:** N. Arians, K. Lindel, J. Krisam, K. Herfarth, D. Krug, S. Akbaba, J. Oelmann-Avendano, J. Debus

**Affiliations:** 1 National Center for Radiation Research in Oncology (NCRO), Heidelberg Institute for Radiation Oncology (HIRO), Heidelberg, Germany; 2 Department of Radiation Oncology, University Hospital Heidelberg, Im Neuenheimer Feld 400, D-69120 Heidelberg, Germany; 3 Heidelberg Ion Beam Therapy-Center, Heidelberg, Germany; 4 Institute for Medical Biometry and Informatics, University of Heidelberg, Heidelberg, Germany; 5 Department Department for Radiation Oncology, Municipal Hospital Karlsruhe gGmbH, Moltkestraße 90, 76133 Karlsruhe, Germany

**Keywords:** Gynecologic cancer, Cervical cancer, Endometrial cancer, Proton therapy, Toxicity, Quality of life

## Abstract

**Background:**

The prognosis for patients with cervical or endometrial cancer has improved over the last decades. Thus, reducing therapy-related toxicity and impact on quality of life have become more and more important. With the development of new radiotherapy techniques like IMRT (Intensity-modulated radiotherapy) the incidence of acute and chronic toxicities has already been reduced. Nevertheless, rates of complications requiring medical treatment range from 0.7–8% according to literature. 7.7% of patients develop severe complications after 5 years with an increasing risk for complications of 0.3%/year. Particularly, the volume of the small and large bowel receiving low doses (15 Gy) has been shown to be a predictive factor for the development of higher bowel toxicity. With the introduction of proton therapy into clinical practice, there are new opportunities for optimization of organ at risk-sparing thus possibly reducing toxicity.

**Methods/design:**

The APROVE study is a prospective single-center one-arm phase-II-study. Patients with cervical or endometrial cancer after surgical resection who have an indication for postoperative pelvic radiotherapy will be treated with proton therapy instead of the commonly used photon radiation. A total of 25 patients will be included in this trial. Patients will receive a dose of 45–50.4 GyE in 1.8 GyE fractions 5–6 times per week using active raster-scanning pencil beam proton radiation. Platinum-based chemotherapy can be administered if indicated. For treatment planning, rectum, sigma, large and small bowel, bladder and femoral heads are defined as organs at risk. The CTV is defined according to the RTOG consensus guidelines.

**Discussion:**

The primary endpoint of the study is the evaluation of safety and treatment tolerability of pelvic radiation using protons defined as the lack of any CTC AE Grade 3 or 4 toxicity. Secondary endpoints are clinical symptoms and toxicity, quality of life and progression-free survival. The aim is to explore the potential of proton therapy as a new method for adjuvant pelvic radiotherapy to decrease the dose to the bowel, rectum and bladder thus reducing acute and chronic toxicity and improving quality of life.

**Trial registration:**

Registered at https://clinicaltrials.gov, ClinicalTrials.gov Identifier: NCT03184350, registered 09 June 2017, enrolment of the first participant 19 June 2017.

## Background

Cervical cancer was once one of the most common causes of cancer death for women. With the implementation of regular screenings including Pap smear rates of cervical cancer have fallen by 75% in the United States [1]. Nowadays, cervical cancer contributes nearly 8% of all cancers in women worldwide [2]. The American Cancer Society estimates about 12,820 new cases of invasive cervical cancer for 2017 [3]. Treatment of cervical cancer depends very much on stage at time of diagnosis. Early stages are mostly treated with surgery, whereas patients with FIGO stage IIb or higher should be treated with definitive radiochemotherapy. There are also cases where postoperative radiotherapy is indicated to reduce the risk of tumor recurrence [4, 5]. Risk factors for local recurrence are positive resection margins, positive lymphonodal status, >1/3 stromal invasion, capillary lymphatic space involvement, and large clinical tumor diameter. In these cases, postoperative radiotherapy can improve local-recurrence-free survival from 79 to 88% [5], however resulting in higher toxicity rates than after surgery alone. Further studies showed that simultaneous radiochemotherapy is superior to radiation alone both in the adjuvant [6] and definitive setting [7]. For example, Morris et al. could show improved progression-free survival (80% vs. 63%) as well as overall survival (81% vs. 71%) after combined platinum-based radiochemotherapy compared to radiotherapy alone [8].

Endometrial cancer contributes nearly 5% of all cancers in women worldwide [2]. In Europe and the United States, it is the most common cancer of the female reproductive organs. The American Cancer Society estimates about 61,380 new cases of endometrial cancer for 2017 [3]. Most patients with endometrial cancer are diagnosed in an early stage resulting in a low cancer specific mortality. Primary treatment consists of radical surgery. The prognosis of patients with endometrial cancer depends very much on tumor stage, grading, histology, depth of myometrial invasion, capillary lymphatic space involvement and age. All patients with clear-cell or serous adenocarcinoma histology should receive postoperative percutaneous pelvic radiotherapy. But also patients with endometroid adenocarcinoma FIGO stage Ib or higher and presence of risk factors should receive postoperative radiotherapy [9–11].

Therapy-associated toxicity is the limitating factor for adjuvant pelvic radiotherapy. Recurrence of the vaginal cuff is the most common site of locoregional recurrence for patients with endometrial cancer. With the use of endovaginal brachytherapy, sufficiently high doses for a good local control can be achieved with better sparing of organs at risk than with external beam radiotherapy alone [11]. New technical developments in the field of radiotherapy like IMRT (intensity-modulated radiotherapy), VMAT (volumetric arc therapy) and Tomotherapy have resulted in better sparing of organs at risk (OARs) like rectum, bowel and bladder thus reducing toxicity of pelvic radiotherapy. Nevertheless, rates of complications requiring medical treatment range from 0.7–8% according to literature [12–15]. Especially chronic side effects have a high impact on patients’ quality of life. In a trial with 1784 patients receiving definitive radiotherapy for FIGO stage Ib cervical cancer, 7.7% developed severe complications after 5 years. The risk for developing complications increased for 0.3% per year resulting in an actuarial 20-year-complication rate of 14%. Side effects most commonly consist of gastro-intestinal/gastro-urogenital symptoms like proctitic symptoms, bleeding, fistula, ulcers, hematuria or vaginal ulcers and fistulas [16]. The rate of toxicities correlates with dose per fraction, cumulative dose as well as the volume of the organ at risk affected by radiotherapy. A recent study by Chopra et al. showed that particularly the extent of the bowel being exposed to low doses is a crucial factor for the development of chronic gastrointestinal toxicities [17]. The volume of the bowel receiving less than 15 Gy could be identified as an independent predictive factor for the development of > grade 3 chronic intestinal toxicities. The volume of the small bowel and large bowel receiving 15 Gy or less should be restricted to <257cm^3^ and <250cm^3^, respectively, to avoid late side effects [17].

In the last decade, the use of particles for radiation of certain tumors has found its way into clinical practice. Proton therapy with its characteristic Bragg peak allows for a more precise radiation with better sparing of OARs thus possibly reducing radiation related toxicities. This facilitates dose escalation, particularly in patients receiving combined-modality therapy, for which toxicity is enhanced. A planning study from 2012 could demonstrate that the use of passive scattering proton therapy (PSPT) or intensity-modulated proton therapy (IMPT) resulted in a statistically significant decrease in dose to the small and large bowel and kidneys, while maintaining excellent planning target volume coverage [18]. Lin et al. published a study in 2016 of 11 patients with post-hysterectomy gynecologic cancer who received pencil beam scanning proton radiation therapy (PBS) to the whole pelvis. One patient (9%) developed grade 3 acute gastrointestinal toxicity, no patient developed ≥ grade 3 genitourinary toxicity. The volume of pelvic bone marrow, bladder, and small bowel receiving 10 to 30 Gy was significantly lower with PBS than with IMRT with good coverage of the target volume [19].

Previous work of our group included plan comparisons of IMRT and PBS proton therapy plans based on CT-scans obtained for radiation treatment planning of patients with cervical or endometrial cancer. It could be shown that with the use of active raster scanning PBS proton radiation the V15 of the bowel could be reduced effectively compared to the IMRT plans (unpublished data).

The aim of this prospective study is to evaluate safety and treatment tolerability of pelvic radiation using protons and to explore if reduced dose to the OARs results in clinically apparent reduced side effects and mitigates the influence of pelvic radiation on quality of life. All in all, we aim to assess the potential of proton therapy as a new method for adjuvant pelvic radiotherapy to decrease the dose to the bowel, rectum and bladder thus reducing acute and chronic toxicity and improving quality of life.

## Methods/design

### Study design

The study is designed as a prospective single-center one-arm phase-II-trial evaluating the clinical feasibility and toxicity of postoperative pelvic radiation using proton therapy. Patients fulfilling the inclusion criteria will be treated with active raster scanning proton radiation up to total doses of 45.0–50.4 GyE in 1.8 GyE single dose fractions. 25 patients are planned to be enrolled in a time period of 2 years.

### Study objectives

The primary objective of the trial is the assessment of safety and treatment tolerability defined as the lack of any CTC AE ≥ grade 3 gastrointestinal or urogenital toxicity or premature treatment abortion.

Secondary endpoints are clinical symptoms and toxicity according to the CTC AE version 4.0. criteria, quality of life assessed with the EORTC-QLQ30/−EN24/−CX24 questionnaires and progression-free survival.

### Trial organization and coordination

The APROVE study has been designed by the study initiators at the Department of Radiation Oncology in cooperation with the Institute of Medical Biometry and Informatics at the University Hospital Heidelberg. The study is carried out by the Department of Radiation Oncology in cooperation with the Heidelberg Ion beam Therapy-center. Statistical analysis is performed by the Institute of Medical Biometry and Informatics at the University of Heidelberg. The overall coordination is performed by the Department of Radiation Oncology at University Hospital Heidelberg. This department is also responsible for the overall trial management, database management, quality assurance including monitoring and reporting.

### Investigators

The study investigators are experienced radiation oncologists specialized in the treatment of patients with gynecologic malignancies. Patients will be recruited and treated by the physicians of the Department of Radiation Oncology of the University Hospital Heidelberg.

### Ethics, informed consent and safety

The final protocol was approved by the ethics committee of the University of Heidelberg, Heidelberg, Germany (Nr: S-155/2016) and by an independent expert group of the German Society for Radio-oncology (DEGRO). This study complies with the Helsinki Declaration in its recent German version, the principles of Good Clinical Practice (GCP) and the Federal Data Protection Act. The trial will also be carried out in keeping with local legal and regulatory requirements. The medical secrecy and the Federal Data Protection Act will be followed. The ClinicalTrials.gov Identifier is NCT03184350.

### Data handling, storage and archiving of data

All findings including clinical and laboratory data will be documented by the investigator or an authorized member of the study team in the subject’s medical record and in the case report form (CRF). The data will be stored and archived according to the §13 of the German GCP-Regulation and §28 c of the German X-Ray Regulation (StrlSchV) for at least 30 years after the trial termination.

### Patient selection

Inclusion criteria according to the protocol are:

- Histologically confirmed cervical or endometrial cancer
- Indication for postoperative radiotherapy
- Karnofsky Index ≥70
- Age between 18 and 80 years
- Written informed consent

Exclusion criteria are the following:

- patients refusal or incapability of informed consent
- implanted active medical devices lacking approval for ion beam radiation
- metallic implants in the radiation field, e.g. hip prostheses
- prior pelvic irradiation
- participation in another clinical trial which might influence the results of the APROVE trial

Simultaneous chemotherapy is NOT an exclusion criterium.

### Sample size calculation and statistical analysis

The APROVE trial is a prospective single-center one-arm phase-II-study. Primary endpoint is the evaluation of safety and treatment tolerability of pelvic radiation using proton beam radiotherapy. 25 patients will be included in the study. Sample size calculation was performed to demonstrate a treatment tolerability rate higher than 80% with a power of 1-β = 0.90 using a binomial test at a one-sided significance level of α = 0.1 under the assumption of an actual treatment tolerability rate of 0.96. The primary analysis includes all enrolled patients. In addition, a per-protocol analysis will be performed. For the primary endpoint, a point estimate for the tolerability rate will be calculated alongside a one-sided 90- and 95%- confidence interval. Methods of descriptive data analysis will be used to evaluate the secondary endpoints and safety data. This includes calculation of appropriate measures of the empirical distribution and graphical display of the results.

### Investigation schedule

#### Indication

The oncological treatment concept for each patient is based on interdisciplinary assessment following approved standard therapies and guidelines. The adjuvant treatment is defined after surgery and complete staging including thoraco-abdominal CT scan and pelvic MRI scan. This allows for histologically confirmed diagnosis, classification and staging.

According to institutional policies the following patients receive whole pelvic radiotherapy:

Cervical cancer patients: All patients with FIGO stage ≥ IIb, N+ or positive resection margins and no possibility of further resection. Furthermore, patients with FIGO stage < IIb and a combination of risk factors like lymphangiosis carcinomatosa (L1), deep stromal invasion, tumor size >4 cm (according to the Sedlis criteria [5] and the german S3-guidelines).

Endometrial cancer patients: All patients with serous, clear cell or undifferentiated histology and all patients with endometroid, mucinous or adenosquamous carcinomas FIGO stage ≥ III. Furthermore, patients with FIGO stage I/II according to the ESMO-ESGO-ESTRO Consensus Conference on Endometrial Cancer [20] taking into account lymphadenectomy and risk factors such as lymphovascular invasion, depth of myometrial invasion, grading and patient age (according to the risk classification developed by the PORTEC and GOG groups).

In general, every patient with an indication for percutaneous pelvic radiotherapy also receives a high dose rate (HDR)-vaginal brachytherapy (VBT)-boost.

#### Radiotherapy-planning

In order to ensure accurate reproducibility of patient positioning, the patient is immobilized using a ProSTEP (ITV, Innsbruck, Austria). CT scans are obtained in the immobilization device in 3 mm-slices. Target volumes and organs at risk are defined using Siemens TPS Version, Syngo RT Planning VC13A for contouring and dose calculation. The target volume is defined according to the RTOG consensus guidelines [21]. A margin of 5 mm and 7 mm in beam direction will be used to create the PTV (planning target volume) according to internal standards. OARs are small and large bowel, sigma, posterior wall of the rectum, bladder and femoral heads. Tolerated maximum doses to OARs must not exceed the TD5/5 for each organ [22].

#### Radiation therapy

After validation of the treatment plan by the radiation oncologist and the physicist in charge, treatment is applied in daily fractions of 5–6 × 1.8 Gy per week to a total dose of 45–50.4 Gy using active raster scanning proton radiation. Total treatment duration hence is 4–5 weeks. Isocenter and patient positioning are checked daily by on-line orthogonal x-ray-imaging and by regularly performed off-line positioning control CT scans. Treatment time is expected to be about 35–45 min. Treatment will be carried out on an out-patient basis unless the patient’s condition requires hospital admission.

HDR-VBT-boost is applied the week after finishing percutaneous proton radiation to minimize the confounder of additional acute bladder and rectal toxicities. HDR-VBT-boost is delivered to the upper two thirds of the vagina by using a vaginal cylinder. Single fractions of 5 Gy to the vaginal mucosa at 5 mm depth are delivered (two fractions, cumulative dose 10 Gy), CT-based treatment planning is used for 3-D-optimization.

#### Monitoring during treatment/adverse events

Patients are evaluated weekly during radiotherapy. Radiotherapy-related toxicities are assessed using the National Cancer Institute (NCI) Common Toxicity Criteria (CTC) version 4.0. Toxicity will be evaluated pre-treatment, weekly during radiation therapy and at follow-up. Unacceptable toxicity for every individual patient is defined as unpredictable or irreversible ≥ grade 4 toxicity. Unacceptable toxicity resulting in premature termination of the whole trial is defined as any grade 5 toxicity, two consecutive grade 4 toxicities or 5 consecutive grade 3 toxicities.

Expectable possible acute toxicities (up to 3 months post radiation therapy) are fatigue, loss of appetite, weight loss, skin toxicity, nausea, vomiting, irritable bowel syndrome, diarrhea, proctitis, dysuria, hematological toxicity. All acute toxicities should resolve within a few weeks after radiation therapy. Late side effects are rare and are defined as symptoms appearing at least 3 months post radiation. These could include chronic diarrhea, malabsorptive syndrome, chronic bladder inflammation, enterocolitis, strictures, fibroses, ulcers, chronic bleeding. Very rare symptoms are fistulation, perforation, peritonitis, intestinal necrosis or ileus necessitating surgical intervention.

#### Follow up

**Fig. 1:**
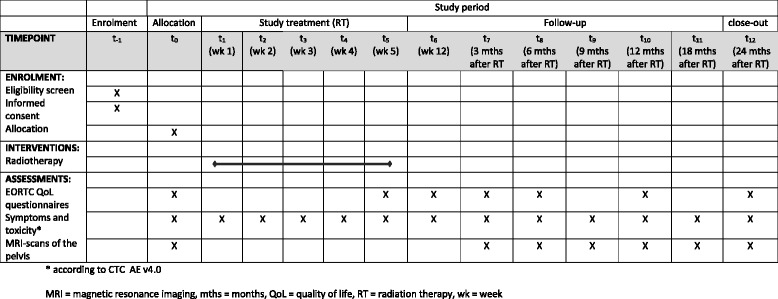
Study schedule

Patients are included into standard gynecology follow-up program. Additionally, regular study visits at 6 weeks, 3 months, 6 months, 9 months, 12 months, 18 months and 24 months post treatment are intended. Each visit includes update of medical history, assessment of symptoms and treatment toxicity. In addition, pelvic MRI-scans are performed 3, 6, 9, 12, 18, 24 months post treatment and quality of life is assessed 6 weeks, 3, 6, 12 and 24 months post treatment (Fig. 1).

### Duration of the study

The primary objective of the study is to prove good treatment tolerability of pelvic radiation using proton beam radiotherapy, defined as the non-occurrence of CTC grade 3 gastrointestinal or urogenital toxicities during radiotherapy and up to 3 months after its completion. To assess the primary endpoint, the final study visit will be 3 months after the last patient completed the radiotherapy. The study ends 2 years after the last patient was treated. Recruitment of the patients is planned over a time period of 2 years.

## Discussion

The prognosis for patients with cervical or endometrial cancer has improved dramatically over the last decades. This is mostly due to more intensive screening programs, better prevention but also improved therapeutic options. Thus, developing new therapeutic techniques in order to reduce therapy-associated long-term toxicities and to improve quality of life of cancer patients has become more and more important. With the development of IMRT the incidence of acute and chronic toxicities could already be reduced. Nevertheless, therapy-associated complications requiring medical treatment are a relevant issue: 7.7% of patients develop severe complications after 5 years with an increasing risk for complications of 0.3%/year [16]. The introduction of proton therapy into clinical practice over the last few years offers one more opportunity for better sparing of organs at risk. A recent study from Philadelphia could already show that also for gynecologic malignancies the use of pelvic proton therapy results in lower doses to OARs with simultaneously good coverage of the target volume [19]. This resulted in a low rate of therapy-associated side effects with only one patient developing acute gastrointestinal toxicity grade 3. This study consisted of only 11 patients. Further studies with bigger collectives are necessary to verify these finding and to evaluate long-term side effects and the impact on quality of life of patients treated with proton therapy for gynecologic malignancies.

## Abbreviations

cc: Cubic centimeters
CT: Computed tomography
DEGRO: German Society for Radio-oncology
ESGO: European Society of Gynaecological Oncology
ESMO: European Society for Medical Oncology
ESTRO: European SocieTy for Radiotherapy & Oncology
FIGO: International Federation of Gynecology and Obstetrics
GCP: Good Clinical Practice
GOG: Gynecologic Oncology Group
Gy: Gray
GyE: Gray-Equivalent
HDR: High Dose Rate
HPV: Human Papilloma Virus
IMPT: Intensity-modulated proton therapy
IMRT: Intensity-modulated radiation therapy
L: Lymphangiosis carcinomatosa
NCI CTC AE v4.0: National Cancer Institute Common Toxicity Criteria version 4.0
OAR: Organ at risk
PBS: Pencil beam Scanning
PORTEC: Post Operative Radiation Therapy in Endometrial Carcinoma
PSPT: Passive Scattering Proton Therapy
PTV: Planning Target Volume
RTOG: Radiation Therapy Oncology Group
TD: Tolerance Dose
VBT: Vaginal Brachytherapy
VMAT: Volumetric Arc Therapy
